# Non-invasive physiological indicators of welfare in dairy cows

**DOI:** 10.1017/awf.2026.10063

**Published:** 2026-02-05

**Authors:** Louise Kremer, Kees van Reenen, Akke Kok, Eddie A.M. Bokkers, Gerrit Gort, Jasper Engel, Joop T.N. van der Werf, Laura E. Webb

**Affiliations:** 1 Animal Production Systems group, Wageningen University & Research, The Netherlands; 2 Wageningen Livestock Research, Wageningen University & Research, PO Box 338, 6700 AH Wageningen, The Netherlands; 3 Wageningen Social and Economic Research, Wageningen University & Research, PO Box 338, 6700 AH Wageningen, The Netherlands; 4 Biometris, Wageningen University & Research, PO Box 16, 6700 AA Wageningen, The Netherlands

**Keywords:** Animal welfare, dairy cattle, hair cortisol, heart-rate variability, milk yield, personality

## Abstract

Indicators of dairy cow welfare are important for the future assessment and improvement of cow welfare on-farm. The objective of this study was to investigate three categories of non-invasive physiological parameters as potential indicators of welfare in dairy cows, namely cumulation of cortisol in the hair, variability in heart rate (HRV), and variability and composition of milk yield, while taking personality traits into account. These indicators were assessed when cows (all primiparous; n = 48) were housed under reference conditions and when exposed to either improving or worsening housing conditions (weekly changes over the course of six weeks). The worsening housing led to an increase in heart rate and a decrease in milk yield. The housing effects on HRV and other milk-derived indicators, however, were affected by the personality traits of activity, fearfulness and sociability. Less active cows, less fearful cows and less social cows all displayed increases in HRV in the improving housing, but more active cows showed against expectations increased HRV in the worsening housing. More fearful cows showed increases in daily milk fluctuations in the worsening housing. These results point to HRV and milk-derived indicators, the latter of which are often routinely collected and that in addition to being non-invasive are also non-intrusive, as providing interesting physiological indicators of dairy cow welfare which will warrant further research.

## Introduction

Indicators of dairy cow (*Bos taurus*) welfare are important for the future assessment and improvement of cow welfare on-farm. Nowadays, many definitions of animal welfare incorporate the notion of affective states, with animal welfare being increasingly defined as some sort of cumulative balance between positive and negative affective states across time (Green & Mellor 2011; Reimert *et al.* 2023; Rault *et al.* 2025). Affective states reflect transient experiences, which may or may not be consciously felt, that are not hedonically neutral (Paul *et al.* 2018) and can be described in a two-dimensional space along the axes of arousal (i.e. low to high activation) and valence (positive/pleasant to negative/aversive) (Mendl *et al.* 2010).

Potential indicators of animal welfare are derived from animal behaviour, cognition or (neuro)physiology, all of which offer a potential window into the minds of non-verbal beings. A potential physiological indicator of welfare is hair cortisol which offers an integrative value of retrospective levels of circulating cortisol (Meyer & Novak 2012). It has been shown to increase in humans in response to chronic stress (Stalder *et al.* 2017) and psychiatric disorders (Wosu *et al.* 2013). In cows, elevated concentrations of hair cortisol have been associated with impaired welfare (e.g. body injuries and dehydration; Sharma *et al.* 2019), although no correlations were found between hair cortisol concentrations and traditional on-farm welfare assessment scores (van Eerdenburg *et al.* 2021).

Other potential physiological indicators of welfare include those reflecting heart-rate variability (HRV), which indicates the response flexibility of the heart to environmental demands (Thayer *et al.* 2009). In both humans and cows, negative states reflective of poor welfare (e.g. depression and chronic stress) have been associated with low resting HRV (Kovacs *et al.* 2015; Sgoifo *et al.* 2015; da Estrela *et al.* 2021), whereas positive states (e.g. cheerfulness and calmness) have been associated with higher resting HRV (Geisler *et al.* 2010; in cows the evidence is more limited: Lange *et al.* 2020; Keeling *et al.* 2021). Although these results are promising, there are also contradictions in both the human and cow literature (Ede *et al.* 2019). For example, a decreased HRV was linked to human ‘joy’ (Kreibig 2010) and an increased HRV was linked to cow lameness (Kovacs *et al.* 2015).

Finally, routinely collected milk-derived data have been hypothesised as being linked to welfare. Milk yield has been shown to decrease in instances where cow health is impaired (e.g. mastitis; Rajala-Schultz *et al.* 1999) and in response to certain stressors (e.g. mixing; Phillips & Rind 2001). Recent studies in dairy cows showed that lower daily fluctuation in milk yield (i.e. day-to-day variation in milk yield) and lower fat content were associated with an increased ability to recover from challenges, i.e. resilience (Poppe *et al.* 2020, 2021). While the underlying mechanisms behind individual resilience remain largely unknown (Southwick & Charney 2012; Southwick *et al.* 2014), empirical studies have highlighted the role of psychological factors in one’s ability to respond to adversity (De la Fuente *et al.* 2021). Considering their involvement in processes related to individual ability to recover from challenges, fluctuation in milk yield and composition might thus also convey information relative to cow welfare.

Indicators of animal welfare can be mediated by personality traits. Personality is commonly defined as “behavioural differences between individuals that are consistent over time and across situations” (Réale *et al.* 2007). Personality has been linked to, for example, the development of stereotypies (e.g. Mason & Latham 2004; Ijichi *et al.* 2013), interaction with ‘enriching’ objects (Meira *et al.* 2023), learning and problem-solving (e.g. Overington *et al.* 2011; Webb *et al.* 2015; Wat *et al.* 2020) and optimism as assessed in judgment bias tests (e.g. Asher *et al.* 2016), as well as physiological patterns in the hypothalamic pituitary adrenal (HPA) axis and autonomic nervous system (e.g. Koolhaas *et al.* 1999; van Reenen *et al.* 2005; Krause *et al.* 2017). These findings highlight the importance of taking personality into account when attempting to identify indicators of animal welfare.

The objective of this study was hence to investigate these three categories of non-invasive physiological parameters as potential indicators of welfare in dairy cows, namely cumulation of cortisol in the hair, variability in heart rate, and variability in milk yield, while taking personality traits into account. We hypothesised that physiological indicators of welfare would vary according to the valence continuum delineated by the housing conditions in a personality-dependent manner.

## Materials and methods

### Ethical approval

This study was carried out at the Dutch experimental farm Dairy Campus in Leeuwarden, The Netherlands, between February 2019 and January 2020. The experiment was approved by the Central Committee on Animal Experiments (The Hague, The Netherlands), approval number AVD4010020174306. All methods involving animals during the study were carried out in accordance with the ‘Wet op de Dierproeven’ (law on animal experiments) and ARRIVE guidelines.

### Study animals and management

The experiment lasted a year and was divided into three batches (four months per batch) of four groups of cows each housed in a separate pen. The four pens within one batch were adjacent and within the same barn. Each pen included eleven lactating and pregnant Holstein-Friesian cows, with four primiparous cows (focal cows) and seven multiparous companion cows. This resulted in an experimental design of four pens with four focal cows per batch (n = 16 per batch) and a total of 48 focal cows across the entire experiment. Focal cows were pseudo-randomly allocated to their group to balance for their days in milk (mean [± SD]: 165 [± 5.5] days), milk production (25.2 [± 0.6] kg) and bodyweight (606 [± 6.0] kg). Companion cows were pseudo-randomly allocated to their group to balance for their parity (3 [± 0.1]), milk production (30.4 [± 0.7] kg) and bodyweight (707 [± 7.4] kg). All cows were healthy and confirmed pregnant at the beginning of the experiment. One focal cow in batch 3 was replaced during the second week of the experiment due to miscarriage (negative treatment). Solid partitions were installed between the pens to prevent visual and tactile contact between the groups. Every morning around 0700h, each pen was delivered a total mixed ration of maize silage (35%; percentage based on dry matter), grass silage (30%), concentrates (20%), ground whole soy (10%), ground whole wheat (3%) and minerals (2%). Additionally, each pen had free access to one water trough and to one automatic concentrate dispenser delivering a pre-determined daily amount of concentrates based upon individual milk production. All cows were milked twice a day around 0500 and 1500h.

**Figure 1.**
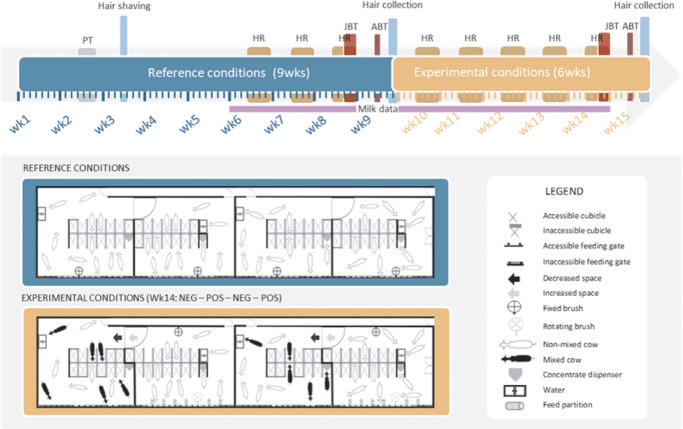
Schematic representation of the overall experimental design to which the dairy cows were exposed, revealing (a) timeline of the data collection and (b) schematic layout of the four adjacent study pens in the reference and experimental conditions (adapted from Kremer *et al*. 2021). ABT: attention bias test, HR: heart rate, JBT: judgment bias test, NEG: negative housing, POS: positive housing, PT: personality tests. ABT and JBT data are described elsewhere (Kremer *et al*. 2021).

**Figure 2.**
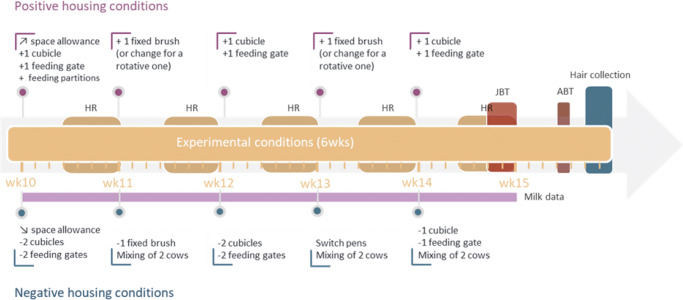
Timeline of the detailed housing changes performed in the experimental conditions applied to the dairy cows. HR: Heart rate data collection, JBT: judgment bias test (testing moment), ABT: attention bias test. In the present study, JBT and ABT results are not presented, as they are described in our sister article (Kremer *et al*. 2021).

The overall experimental design is clarified in Figure 1. In each batch, cows were first housed under reference conditions (9 weeks), before being housed under one of two experimental conditions (6 weeks): weekly-improved (hereafter ‘positive’; n = 24 cows in six pens) or weekly-worsened (hereafter ‘negative’; 24 cows in six pens) housing conditions. In the reference phase, each group had access to exactly 11 cubicles, 11 feeding gates and one fixed brush, hence a 1:1 ratio of cubicles/gates to cow. The social composition of the group was kept constant during this phase. In the experimental phase, which started in study week 10, changes were implemented every Friday after the afternoon milking to create a negative or positive housing situation. Housing changes are described in detail in Figure 2. The aim was to induce positive and negative shifts in the background affective states of cows, and subsequently welfare, by exposing them to an accumulation of either positive or negative events. Contrasted housing conditions are commonly used and accepted in models of chronic mild stress in rodents, and are thought to prevent habituation to any one change. Chronic mild stress models all involve the ‘chronic administration of a variety of stressors’, with stressors being presented predictably or not over time in laboratory rodents (Willner 2017). Providing novel resources and objects to improve animal welfare has been linked to habituation over time leading to a decrease in their effects for both assumed positive and negative stimuli (Waynert *et al.* 1999; Bowman *et al.* 2015). The events applied in the present study revolved around three housing facets which are known to influence cow welfare: the stocking density (Fregonesi *et al.* 2007; Schütz *et al.* 2015; Winckler *et al.* 2015), the social stability within the groups (Schirmann *et al.* 2011; Wilcox *et al.* 2013; Gutmann *et al.* 2015) and the level of ‘enrichment’ (DeVries & Von Keyserlingk 2006; Huzzey *et al.* 2006; McConnachie *et al.* 2018). Note that the term enrichment has been rightly criticised for being used in many different ways (Veissier *et al.* 2024), and in this study should be taken as meaning ‘multiple and varied brushing opportunities’ only. Cows housed in our improving conditions had more space and access to progressively more cubicles and feeding gates, while cows in the worsening conditions had less space and access to progressively fewer cubicles and feeding gates. Additionally, the group composition remained unchanged in the positive conditions, whereas groups in the negative conditions were subjected to frequent mixing. Finally, groups in the positive conditions were provided with additional brushes, while groups in the negative conditions were brush-deprived.

### Personality tests

In week 2 of the reference conditions, focal cows were subjected to three standard challenge tests, namely the open-field, the novel-object and the runway tests. The replacement focal cow in batch 3 (negative treatment) could not be subjected to the personality tests. Focal cows were videotaped (CAMCOLBUL2, Velleman, Belgium) for the open-field and the novel object tests, and live-scored for the runway test using a computer equipped with The Observer XT 10 (Noldus Information Technology BV, Wageningen, The Netherlands). Protocols were adapted from previous studies conducted in cattle (van Reenen *et al.* 2004; Gibbons *et al.* 2010) and are explained in Kremer *et al.* (2021). Briefly, during the open-field test, focal cows were brought into an unfamiliar arena, where they remained alone for 10 min. At the end of the open-field test, the novel-object test immediately started, and a novel object was lowered remotely from the ceiling of the arena until it touched the floor. The novel object, which was two orange cones attached together, was then lifted 1 m above the floor and remained in this position for the entire duration of the test, i.e. for 10 min. On the days following the open-field and the novel-object tests, focal cows were subjected to the runway test, which was conducted in a corridor familiar to the cows and leading to the milking parlour. Prior to the test, a group of four focal cows and two companion cows from the same pen were brought to a waiting area for 10 min. The focal cow to be tested was subsequently brought 18 m away from the waiting area and positioned into a starting area, where she could face the rest of her group for 1 min. Thereafter, the metal bar preventing the focal test cow from reaching the rest of the group was removed and the test started for 5 min. To extract personality traits, a Principal Component Analysis (PCA) was conducted following previous recommendations (Budaev 2010), and included a total of six measures to ensure an (animal:parameter) ratio superior to 5: the proportion of time spent in contact with the novel object, the latency to touch the novel object, the proportion of time spent in contact with the walls during the open-field and novel-object tests, the proportion of time spent in locomotion during the open-field and novel-object tests, and the proportion of time spent less than 2 m away from the group during the runway test. Data were log-, square- or logit-transformed to achieve approximate normality (van Reenen *et al.* 2004; Webb *et al.* 2015).

### Physiological measures

Physiological indicators of welfare are linked to a number of important limitations (Kremer *et al.* 2020). First, they may reflect affective arousal rather than valence, and any study of physiological changes linked to welfare should make sure to control for arousal (Ede *et al.* 2019). In the present study, we included measures of heart rate (HR) to compare arousal between treatments, and corrected for HR in our HRV analyses, see *Statistical analysis.* Second, the circadian rhythms and response timing of indicators should be understood or at least accounted for. It was not within the scope of the present study to also assess daily variations of our indicators, however, we ensured that these indicators were collected at the same time each day (the timing for HRV collection was not entirely possible to control, see *Heart rate variability indicators*). Third, as with other types of indicators, it is important that studies distinguish between state (i.e. modulated by transient internal or external events) and trait (i.e. determined by the animal’s genetics and early life experiences) physiological differences, which is possible with longitudinal studies controlling for individual differences, i.e. personality. Fourth, invasive (defined here as the introduction of instruments into the body or body cavities) and intrusive (defined here as causing disruption or annoyance) sampling to collect physiological data is not preferred as it may impact the welfare of animals, and non-invasive and non-intrusive sampling should be favoured. However, although all of our indicators could be non-invasively collected, two of our indicator categories, namely hair cortisol and HRV, were intrusive, see details below.

#### Hair cortisol

Hair was shaved along the left side of the focal cows’ vertebral column using an Aesculap Favorita CL hair clipper (Aesculap, Germany) and was discarded on week 4. The clipper was dusted off between each cow. Hair from the dominant colour of the focal cow in the shaven area was then collected at the end of both reference and experimental phases in week 9 (n = 48) and 15 (n = 48). Therefore, the hair collected in the reference phase was the hair grown during the last six weeks of the reference phase, and the hair collected in the experimental phase was the hair grown during the six weeks of the experimental phase. Hair samples were then stored in darkness in zip-lock plastic bags at –20°C until assays. Hair samples were prepared and analysed at the Dutch Animal Health Service (Royal GD, Deventer, The Netherlands) based on a protocol adapted from previous studies (Koren *et al.* 2002; Burnett *et al.* 2014). Hair samples were washed three times with decanted isopropanol and dried during approximatively 24 h. Samples were then cut with clean scissors and ground into powder using a Retch grinding cup with a 20 mm bead in a Retch beater (Retch, Haan, Germany) for 5 min. After grinding, approximately 200 mg of hair powder were transferred into glass tubes wherein methanol was added and mixed with the powder. Tubes were thereafter sonicated for 30 min, placed in an oven for about 24 h and centrifuged. After centrifugation, a solution of 4 mL supernatant was collected from each tube and its content in methanol was evaporated using nitrogen. Subsequently, 500 μL of physiological saline solution was added to each tube and mixed with the tube content. Tubes were then placed in an ultrasonic bath for 10 min and the solution was mixed. Cortisol concentration was then measured by chemiluminescence on an Immulite 1000 plus with the associated test kit for cortisol analysis (LKCO1 Siemens, Germany). Intra- and inter-assay coefficients of variability were of 6.5 and 11.2%, respectively.

#### Heart-rate variability indicators

The impact of HR and activity on HRV (Ede *et al.* 2019) was accounted for by only assessing HRV in resting animals, while controlling for HR. Focal cows were habituated to wearing Zephyr Bioharness^TM^ HR belts (Zephyr Technology Corporation, USA) for a minimum of 1 h, 1 h 30 min and 2 h for three days in study weeks 4 and 5. In study weeks 6 to 8 of the reference phase and 10 to 14 of the experimental phase, HR data were collected once a week for each focal cow. On recording days (Tuesday to Friday), the HR data of four cows were measured from 0830 to 1500h. This sampling time-frame was utilised to ensure that the HR measures would not simply reflect cows’ acute responses to the changes in the housing conditions (which occurred on Friday afternoons). Cows were pseudo-randomly attributed to one day of recording to ensure that: (1) HR measurements were obtained from one cow per group; and (2) the focal cows were not planned for judgement bias training or testing (Kremer *et al.* 2021), on that specific day. For practicality, the weekly order of cows for HR recordings was kept constant during the experiment.

Heart rate belts were adjusted to the body of the cows using rubber straps. Belts were attached around the cow’s thorax when she was either locked at the feeding gates or standing up in a cubicle. After securing the belts, the electrode sites were covered with ultrasound transmission gel for optimal electrode-skin contact. A pilot study was conducted to identify the optimal positions of the two electrodes on the thorax. Eventually, the electrodes were positioned above the left elbow joint and on the sternum. An accelerometer tag (IceQube, IceRobotics, South Queensferry, UK) was also attached to the left hind fetlock joint to synchronise HR data with lying bouts, since posture and activity are known to influence HRV measures (Hagen *et al.* 2005).

The RR-interval data, which represent the time-intervals between the peaks of two consecutive heartbeats, were exported and analysed using Kubios HRV Premium 3.5.0 (Kubios, Kuopio, Finland). The noise detection level was set at medium and the remaining artefacts were corrected using the automatic algorithm correction that was previously validated (Lipponen & Tarvainen 2019). Heart rate measures were calculated for every 5-min time window of each RR signal stream. The measures selected for statistical analyses are described in Table 1. Frequency-domain measures were estimated from the detrended RR series using Fast-Fourier Transformation. Heart-rate variability frequency bands were set following the recommendations of Von Borell *et al.* (2007). Non-linear measures were estimated using Pointcaré plot. Finally, only time-windows during which cows were lying down and which contained less than 5% of corrected beats were included in the statistical analyses, as previously advised (Von Borell *et al.* 2007). The number of valid time-windows per focal cow per housing condition ranged from 6 to 184 per cow for the HR measures, with 2,005 useable 5-min windows for cows in the negative treatment and 2,412 useable 5-min windows for cows in the positive treatment. This variation in available time-windows for HR data was an unavoidable consequence of our specific methods. For analyses, all HR measures were averaged across both the reference and the experimental conditions for each cow.

**Table 1.** Definitions and units of the heart rate and heart-rate variability measures selected for this study on dairy cows (adapted from Shaffer & Ginsberg 2017)

Heart rate measure	Units	Definition
*Time-domain*		
HR	beats.min^–1^	Heart rate, i.e. the number of heart beats per minute
*Frequency-domain*		
LF	%	Relative power of the low frequency band (0.050–2 Hz)
HF	%	Relative power of the high frequency band (0.2–0.58 Hz)
*Non-linear*		
SD1	ms	Poincaré plot standard deviation perpendicular the line of identity. SD1 is equal to RMSSD, i.e. the root mean square of successive RR interval differences
SD2	ms	Poincaré plot standard deviation along the line of identity
SD1.SD2^–1^	n.u	Ratio of SD1 to SD2
SampEn	n.u	Sample entropy, which measures the regularity and complexity of time series

#### Milk-derived measures

From study week 6 onwards, milk yield (MY) was automatically recorded at the individual level during each milking and subsequently averaged across days and study weeks (except for weeks 9 and 15). Additionally, 10 mL milk samples were collected four times a week in tubes containing Bronopol as a preservative to assess fat, protein and lactose composition (ISO, 2013; Qlip, Zutphen, The Netherlands).

### Statistical analysis

All statistical analyses were performed using R 4.0.5 (R CORE Team 2015). Through the different stages of the experiment, the first author (LK), who was the one to perform the statistical analyses, was aware of the group allocation to the housing conditions but unaware of the personality scores of the focal cows until personality data were analysed. Other authors remained blind to treatment and cow personality.

#### Personality scores

All behavioural variables were analysed using The Observer XT 10. The outcomes of the behavioural tests and PCA that were used to identify the personality traits of focal cows are described in Kremer *et al.* (2021). The PCA was performed on the correlation matrix and only three rotated components (RC) were retained based on the fact that they explained more than 75% of the total variance. Interpretation of the RC was exclusively based on loadings rated ‘excellent’, (i.e. with an absolute value superior to 0.71) (Comrey & Lee 2013), and revealed that cow personality (in the contexts used in this study) could be characterised in a three-dimensional space. The first RC loaded high on number of locomotory bouts, time spent in locomotion and time spent in contact with walls and was labelled ‘activity’. The second RC loaded high on time spent in contact with novel object and low on latency to touch novel object and was labelled ‘fearfulness’. The third RC loaded high on time spent within 2 m from group and was labelled ‘sociability’.

Cow scores on each of these three personality traits were extracted from the PCA. For each personality trait, cows were, thereafter, classified into two classes depending on the median score of the considered trait (activity: –0.08, fearfulness: 0.23, sociability: 0.26). The distribution of cows in the different housing conditions according to their personality traits is detailed in Table S1 (see Supplementary material). Cows were relatively well divided between the positive and negative housing treatments according to their activity and fearfulness traits, but not according to their sociability trait (i.e. 10 and 14 social cows were subjected to the positive and negative treatments, respectively; while 14 and 9 non-social cows were subjected to the positive and negative treatments, respectively).

#### Milk-derived measures

Weekly fat- and protein-corrected milk (FPCM) was calculated using the MY and the milk composition of each study week based on the following formula (CVB 2012):

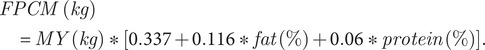

Furthermore, one value of milk persistency (kg per day) was calculated for each focal cow based on daily MY during both the reference (week 6 to 8) and the experimental periods (week 10 to 14); and it was defined as the slope of the lactation curve. Here, the lactation curve was modelled as a straight line since milk decline is said to be linear past the lactation peak (Wilmink 1987). The slope of the lactation line was determined for each focal cow from the linear regression modelling MY according to the days of the aforementioned weeks. Persistency reflects how much the production of milk declines over time, or rather the ability to maintain high levels of milk yields past the peak production. Our expectation was that milk production would decline faster for cows under the negative treatment. Finally, fluctuations in daily MY were assessed by calculating the log-transformed variance (LnVar) of daily MY deviation from the fitted lactation curves, i.e. from the log-transformed variance of the residuals extracted from the fitted curve (Poppe *et al.* 2020). For each cow, daily MY fluctuations in the reference and in the experimental periods were assessed separately – but from the same line used to obtain milk persistency. LnVar is a measure of how much milk production fluctuates, in a sense of how much it deviates from the predicted lactation curve of the individual (based on its past production). Further milk-derived measures included the percent fat (pFat), protein (pProt) and lactose (pLac) in the milk.

#### Regression analysis

Regression analyses were used to assess the influence of the housing conditions and personality scores on a pre-selected set of non-invasively collected physiological measures. Analyses were conducted using Linear Mixed Models (LMMs) and employing lmer from the lme4 package (Bates *et al.* 2015). LF, HF, fat quantity and protein quantity were log-transformed.

Unless specified otherwise, all models included batch, the three personality traits (each expressed as two-level factors according to heifers’ median score on the corresponding trait), the housing conditions (Reference, Positive, Negative) and the two-way interactions between the personality traits and the housing conditions as fixed factors. Random factors included focal cow nested in group/pen, itself nested in batch. Hair colour (black or white) was also included as a fixed factor in the LMM assessing the effect of personality and housing conditions on hair cortisol, since hair colour influences hair cortisol concentrations (Ghassemi Nejad *et al.* 2017). Concerning models of HRV measures, HR was also included as a covariate to control for possible differences in arousal. The interaction effect between HR and housing was tested and dropped from all models, since it was not found to be significant. Finally, for milk persistency, the explanatory variable housing was divided into two factors solely (either positive or negative) to model a single lactation curve per cow between week 6 and 14.

A weighing scheme was applied on HR and milk data to account for the fact that they were averaged from different numbers of observations – depending on (1) the number of valid time-windows per focal cow per housing conditions for the HR measures, and (2) the number of studied weeks per cow per housing conditions (i.e. 3 and 5 for the reference and experimental conditions, respectively) for the milk-derived measures. The weighing scheme was used to account for potential heteroscedasticity, and potential outliers were identified using the compute_redres function from the redress package (Goode *et al.* 2021). Residuals of models with no weight scheme were investigated for normality, linearity and heteroscedasticity using the simulateResiduals function from the DHARMa package (Hartig & Hartig 2017).

As multiple analyses were conducted on the same dataset, only *P* < 0.05 results are reported as significant. For pairwise comparisons on the housing factor (3 comparisons) we adjusted *P*-values by multiplying them by 3. For pairwise comparisons on housing × personality interactions (9 comparisons) we adjusted *P*-values by multiplying them by 9 (Bonferroni adjustment).

## Results

**Figure 3.**
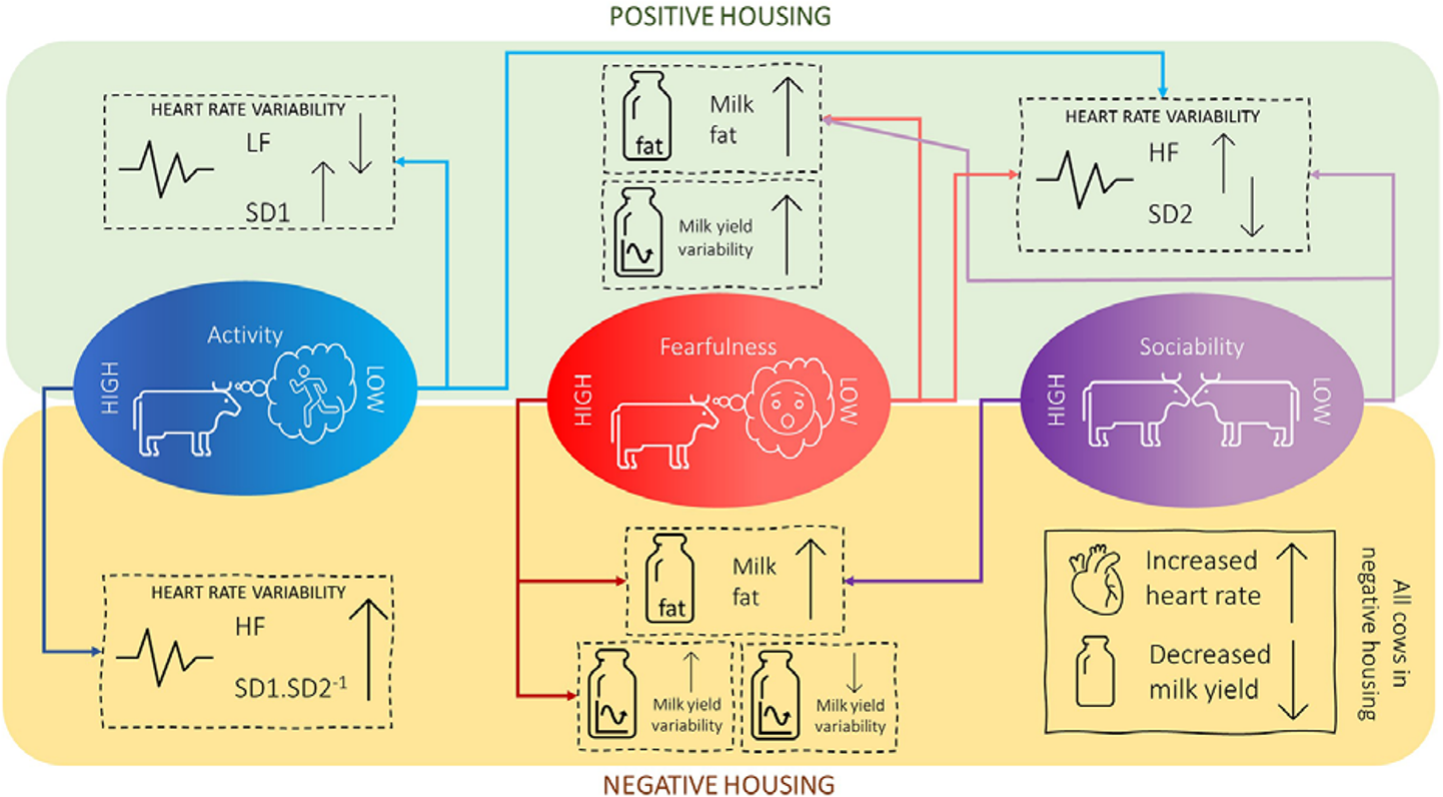
Summary of findings in terms of the effects of housing and personality on the physiology of dairy cows.

The main effects of housing and personality on the different physiological measures are described in Table 2, together with the *P*-values of their interaction effects. The means for the housing × personality interactions that were found to be significant are shown in Table 3. A rough summary of these results is presented in Figure 3. Significant negative relationships were found between HR and five parameters of HRV, namely HF, SD1, SD2, SD1.SD2^-1^ and SampEn (Table 4).

**Table 2.** Effects of housing and personality (raw means ± standard errors) on different physiological measures in dairy cows (n = 48). HC (in ng/g), HR (in beat/min), HF (in %), LF (in %), SD1 (in ms), SD2 (in ms), SD1.SD2^-1^, SampEn, MY (in kg), FPCM (in kg), Persistency (in kg/day), LnVar (in kg), pFat (in %), pProt (in %), pLac (in %). NA^1^: for milk persistency only, housing had two levels (i.e. either positive or negative) since one single value of persistency was calculated from wk5-wk8 of the reference period to wk10-wk15 of the experimental period

Measures	Housing				Activity		
	Negative	Reference	Positive	*p*-value	Active	Inactive	*p*-value
HC	33 ± 3.1	31 ± 2.4	32 ± 2.6	0.487	28 ± 2.4	35 ± 2.0	0.606
HR	82.3 ± 0.84^a^	79.1 ± 0.72^b^	79.5 ± 1.43^b^	<0.001*	80.5 ± 0.92	79.5 ± 0.67	0.812
LF	39.2 ± 1.33	40.2 ± 0.92	37.5 ± 1.24	0.996	38.2 ± 0.97	40.3 ± 0.86	0.011*
HF	9.6 ± 1.66	9.3 ± 0.97	12.0 ± 1.84	0.398	11.2 ± 1.26	9.0 ± 0.94	0.012*
SD1	6.3 ± 0.81	6.8 ± 0.71	7.7 ± 1.52	0.555	7.4± 0.96	6.4 ± 0.60	0.503
SD2	19.7 ± 0.98	21.0 ± 0.99	20.3 ± 1.74	0.193	20.4 ± 1.01	20.6 ± 0.98	0.819
SD1.SD2^–1^	0.32 ± 0.030	0.31 ± 0.017	0.34 ± 0.0299	0.140	0.34 ± 0.022	0.30 ± 0.016	0.201
SampEn	1.29 ± 0.037	1.34 ± 0.029	1.36 ± 0.038	0.942	1.34 ± 0.025	1.32 ± 0.030	0.698
Milk yield	22.8 ± 0.63^a^	23.7 ± 0.58^b^	23.4 ± 0.88^ab^	0.019*	23.7 ± 0.66	23.1 ± 0.45	0.095
FPCM	25.6 ± 0.65	25.8 ± 0.56	25.9 ± 0.83	0.626	26.3 ± 0.63	25.2 ± 0.44	0.062
Persistency	–0.021 ± 0.010	NA^1^	–0.014 ± 0.009	0.719	–0.031 ± 0.010	–0.004 ± 0.008	0.102
LnVar	0.71 ± 0.22	0.35 ± 0.101	0.49 ± 0.159	0.065	0.52 ± 0.131	0.44 ± 0.111	0.967
pFat	4.86 ± 0.099^a^	4.63 ± 0.072^b^	4.78 ± 0.108^a^	0.036*	4.79 ± 0.073	4.66 ± 0.073	0.502
pProt	3.76 ± 0.046	3.68 ± 0.042	3.68 ± 0.045	0.659	3.75 ± 0.038	3.65 ± 0.035	0.264
pLac	4.44 ± 0.019	4.50 ± 0.015	4.49 ± 0.022	0.054	4.48 ± 0.017	4.48 ± 0.013	0.610

Measures	Fearfulness			Sociability			Hous:Act	Hous:Fear	Hous:Soc
	Fearful	Non-fearful	*p*-value	Social	Non-social	*p*-value	*p*-value	*p*-value	*p*-value
HC	31 ± 2.0	33 ± 2.4	0.780	33 ± 2.5	31 ± 1.9	0.383	0.026*	0.825	0.843
HR	79.2 ± 0.59	80.8 ± 0.97	0.332	79.4 ± 0.77	80.6 ± 0.83	0.600	0.473	0.084	0.308
LF	40.0 ± 0.90	38.5 ± 0.97	0.090	38.7 ± 0.94	39.9 ± 0.90	0.406	0.047*	0.140	0.893
HF	9.1 ± 0.57	11.1 ± 1.49	0.288	11.2 ± 1.36	8.9 ± 0.74	0.199	0.045*	0.011*	0.047*
SD1	6.3 ± 0.36	7.6 ± 1.08	0.592	7.9 ± 1.03	5.9 ± 0.37	0.573	0.019*	0.542	0.813
SD2	20.3 ± 0.91	20.7 ± 1.09	0.911	21.1 ± 1.12	19.9 ± 0.3	0.997	0.344	0.011*	0.014*
SD1.SD2^–1^	0.31 ± 0.010	0.33 ± 0.026	0.473	0.34 ± 0.024	0.30 ± 0.012	0.238	0.010*	0.031*	0.153
SampEn	1.37 ± 0.023	1.29 ± 0.032	0.262	1.33 ± 0.030	1.33 ± 0.025	0.815	0.161	0.036*	0.116
Milk yield	23.8 ± 0.635	23.06 ± 0.468	0.999	22.87 ± 0.527	24.00 ± 0.588	0.422	0.217	0.066	0.805
FPCM	26.0 ± 0.620	25.56 ± 0.438	0.811	25.62 ± 0.517	25.95 ± 0.566	0.870	0.207	0.188	0.626
Persistency	–0.021 ± 0.012	–0.0137 ± 0.007	0.531	–0.017 ± 0.008	–0.012 ± 0.012	0.530	0.426	0.060	0.673
LnVar	0.63 ± 0.124	0.32 ± 0.114	0.099	0.42 ± 0.110	0.53 ± 0.132	0.169	0.110	0.001*	0.963
pFat	4.67 ± 0.081	4.79 ± 0.064	0.569	4.86 ± 0.069	4.59 ± 0.073	0.020*	0.303	0.028*	0.036*
pProt	3.72 ± 0.040	3.68 ± 0.035	0.842	3.76 ± 0.039	3.64 ± 0.034	0.313	0.695	0.529	0.923
pLac	4.49 ± 0.015	4.48 ± 0.015	0.323	4.46 ± 0.014	4.50 ± 0.015	0.695	0.269	0.140	0.502

* *P*-values < 0.05. Hous:Act is the interaction effect between housing and activity, Hous:Fear is the interaction effect between housing and fearfulness and Hous:Soc is the interaction effect between housing and sociability. HC: Hair cortisol, HR: heart rate, HF: relative power of the high-frequency band, LF: relative power of the low frequency band, SD1: standard deviation perpendicular the line of identity in the Pointcaré plot, SD2: standard deviation along the line of identity in the Pointcaré plot, SD1.SD2^-1^: ratio between SD1 and SD2, SampEn: sample entropy, MY: milk yield, FPCM: fat and protein-corrected milk yield, Persistency: milk persistency, LnVar: daily milk fluctuation, pFat: fat content in milk, pProt: protein content in milk, pLac: lactose content in milk

**Table 3.** Raw means (± standard error) for the housing × personality interactions that were found to be significant (*P* < 0.05) in dairy cows (n = 48). Means with no common superscript within an indicator and personality category (a,b,c) or within an indicator and housing condition (x,y) differ significantly (*P* < 0.05). HC (in ng/g), HF (in %), LF (in %), SD1 (in ms), SD2 (in ms), SD1.SD2^-1^, SampEn, LnVar (in kg) and pFat (in %)

	Indicator	Personality category						*P*-value
		High^1^ (Active, Fearful, Social)			Low (Inactive, Non-Fearful, Non-Social)			
		Negative	Reference	Positive	Negative	Reference	Positive	
Housing x Activity	Hair cortisol^2^	29.2 ± 4.57	26.1 ± 3.78	30.4 ± 3.73	36.5 ± 4.05	35.8 ±2.82	32.7 ± 3.82	0.026
	LF	37.5 ± 1.67	39.3 ± 1.47	36.7 ± 1.93	41.1 ± 2.03^ab^	41.2 ± 1.12^a^	38.1 ± 1.67^b^	0.047
	HF	12.6 ± 2.91^a^	9.6 ± 1.36^b^	12.8 ± 3.21^ab^	6.2 ±0.62^ab^	9.0 ± 1.40^b^	11.3 ± 2.16^a^	0.045
	SD1	7.6 ± 1.44^a^	7.0 ± 1.17^ab^	8.0 ± 2.88^b^	4.9 ± 0.44	6.6 ± 0.85	7.4 ± 1.53	0.019
	SD1.SD2^–1^	0.38 ± 0.051^ax^	0.32 ± 0.026^b^	0.34 ± 0.053^b^	0.25 ± 0.013^y^	0.30 ± 0.023	0.34 ± 0.034	0.010
Housing x Fearfulness	HF	9.6 ± 1.24	9.2 ± 0.91	8.2 ± 0.79	9.5 ± 3.05^ab^	9.4 ± 1.76^a^	16.5 ± 3.52^b^	0.011
	SD2	19.6 ± 1.53	20.8 ± 1.45	20.0 ± 1.65	19.8 ± 1.31^ab^	21.2 ± 1.37^a^	20.6 ± 3.36^b^	0.011
	SD1.SD2^–1 2^	0.32 ± 0.020	0.31 ± 0.015	0.29 ± 0.016	0.31 ± 0.056	0.31 ± 0.032	0.40 ± 0.058	0.031
	SampEn^2^	1.4 ± 0.04	1.4 ± 0.03	1.3 ± 0.04	1.2 ± 0.05	1.3 ± 0.05	1.4 ± 0.07	0.036
	LnVar	1.33 ±0.350^ax^	0.55 ± 0.136^b^	0.18 ± 0.137^b^	0.14 ± 0.175^y^	0.15 ± 0.142	0.86 ± 0.274	0.001
	pFat	5.0 ± 0.18^a^	4.6 ± 0.10^b^	4.6 ± 0.16^ab^	4.8 ±0.10^ab^	4.7 ± 0.10^a^	5.0 ±0.12^b^	0.028
Housing x Sociability	HF	10.3 ± 2.54	10.2 ±1.69	14.8 ± 3.72	8.4 ± 1.68^ab^	8.4 ± 0.91^a^	10.0 ±1.64^b^	0.047
	SD2	19.1 ±1.30	21.6 ± 1.58	22.7 ± 3.43	20.6 ±1.51^ab^	20.4 ± 1.19^a^	18.5 ±1.67^b^	0.014
	pFat	5.0 ± 0.13^a^	4.8 ± 0.10^b^	4.9 ± 0.12^ab^	4.6 ± 0.10^ab^	4.5 ± 0.10^a^	4.7 ± 0.17^b^	0.036

HC: Hair cortisol, HR: heart rate, HF: relative power of the high-frequency band, LF: relative power of the low frequency band, SD1: standard deviation perpendicular the line of identity in the Pointcaré plot, SD2: standard deviation along the line of identity in the Pointcaré plot, SD1.SD2^-1^: ratio between SD1 and SD2, SampEn: sample entropy, LnVar: daily milk fluctuation, pFat: fat content in milk.

1 For clarity, ‘Fearful’ is considered the high end of the Fearfulness trait, even though fearful cows showed the lowest loadings on the PCA.

2 Further pairwise comparison did not yield any significant effects, which is why these rows have no superscripts.

**Table 4.** Regression coefficients (ß), standard errors (in brackets) and *P*-values from mixed models testing the effect of heart rate as covariate on different heart rate variability measures taken from dairy cows

Response variables	Heart rate	
	Estimate (standard error)	*p*-value
LF	ß = 0.01 (0.006)	0.075
HF	ß = – 0.08 (0.012)	<0.001*
SD1	ß = – 0.05 (0.008)	<0.001*
SD2	ß = – 0.02 (0.006)	0.004*
SD1.SD2^–1^	ß = – 0.03 (0.006)	<0.001*
SampEn	ß = – 0.01 (0.003)	<0.001*

* *P*-values below 0.05.

### Effects of housing independent of personality

The negative housing led to an increase in HR (*P* < 0.001) and a decrease in MY (*P* = 0.019).

### Active/inactive personality

When inactive cows were transitioned to the positive housing they displayed lower LF as well as higher HF, which could point to higher HRV. When active cows were transitioned to the negative housing, they displayed, against expectations, changes that are concurrent with an increase in HRV: i.e. an increase in HF and SD1.SD2^-1^. In line with this, SD1 was higher in active cows in the negative housing than in active cows in the positive housing (based on model estimates, not raw means), and SD1.SD2^-1^ was higher in active cows in the negative housing compared to inactive cows in the same housing. So, although active cows may have displayed overall higher HRV, they responded against expectations to the negative housing, which seems to have led to an even higher HRV, while inactive cows showed, as we expected, an increase in HRV in response to the positive housing. Although a significant housing × activity effect was found on hair cortisol, pairwise comparisons yielded no significant differences.

### Fearful/non-fearful personality

Cows characterised as having a non-fearful personality profile showed increases in HF, SD1.SD2^-1^, pFat and LnVar, but decreases in SD2 (indicative of long-term HRV) when transitioned to the positive housing. In contrast, fearful cows showed an increase in pFat and LnVar when transitioned to the negative housing. In line with this, LnVar was also higher for fearful cows in the negative housing compared to (1) fearful cows in the positive housing and (2) non-fearful cows in the negative housing. Significant housing × fearfulness effects were found for SD1.SD2-1 and SampEn, but differences disappeared during pairwise comparison. None of the other parameters showed significant changes.

### Social/non-social personality

Cows characterised as having a non-social personality profile showed increases in HF and pFat, and a decrease in SD2 when transitioned to the positive housing. Social cows also showed an increase in pFat, but when they were moved to the negative housing. None of the other parameters showed significant changes.

## Discussion

We investigated the potential of three non-invasive categories of physiological parameters as indicators of welfare in primiparous dairy cows exposed to varying housing conditions over a period of six weeks, namely: hair cortisol, heart-rate variability (HRV) and milk yield (MY) fluctuations and composition.

Overall, we found that personality influenced the physiological responses of cows to housing changes in several instances: Activity influenced LF, HF, SD1, and SD1.SD2^-1^; Fearfulness influenced HF, SD2, LnVar and pFat; Sociability influenced HF, SD2, and pFat. These results suggest that there may be a relationship between individual differences, as measured by novelty and social tests, and physiological responses to housing conditions. Considering that our housing changes were applied gradually over time, it is, however, not possible to determine with absolute certainty whether these personality-related physiological changes in response to the housing conditions might instead reflect changes related to pregnancy (or other time-related factors), rather than differences in how (un)pleasantly cows experienced their housing conditions. Differences in personality-related physiological responses to housing conditions may reflect differences in how individuals experience (or use) a specific element of their environment (e.g. cows using the brush for massage purposes or as a bouncing toy) – but these different experiences may have similar values in terms of welfare.

### Hair cortisol

No significant differences in hair cortisol between cows housed in the negative and the positive conditions were found. This result contrasts with previous studies investigating the link between hair cortisol and various facets of animal housing. Both beef cattle and rhesus macaques (*Macaca mulatta*) housed at higher stocking densities had higher hair cortisol levels than counterparts housed at lower stocking densities (Dettmer *et al.* 2014; Schubach *et al.* 2017). Pigs (*Sus scrofa*) housed in barren conditions had higher hair cortisol levels than pigs housed in enriched conditions (Casal *et al.* 2017) – even though the validity of hair cortisol as an indicator of stress in pigs has been challenged (Heimbürge *et al.* 2020a). This difference in results may potentially arise from the nature of the treatment applied in the present study, which is dynamic as opposed to stable in the aforementioned studies. However, other studies highlight the complexity of linking hair cortisol to dairy cow welfare (Kern *et al.* 2024; Botia *et al.* 2025; Lavrijsen-Kromwijk *et al.* 2025).

The interpretation of our results requires caution considering the methodological limitations of our work. First, we cannot rule out the possibility that season had an effect on our measure of hair cortisol. As we opted for a longitudinal approach, the seasonal effect on hair cortisol is confounded with our treatment effect (Heimbürge *et al.* 2020b); which may have masked housing-induced effects on hair cortisol. However, while batch 1 and 2 moved towards warmer temperatures, batch 3 moved towards colder ones, so perhaps our batches to some extent controlled for this. Second, it is worth noting that our hair cortisol may not accurately reflect the expected housing-induced effects due to inadequate hair sampling moments. In our study, hair samples in the experimental conditions were collected at the end of a 5-week housing treatment, whereas a recent study revealed that the optimal time for hair sampling in cows would be within a 4-week period *after* the end of the stressor to ensure that the section of the hair containing stress-induced cortisol has actively regrown (Heimbürge *et al.* 2020a). This would mean that if our initial two weeks were not sufficiently ‘bad’ to be considered as stressors, then we would have benefitted from collecting new hair samples 4 weeks after the end of our study, when conditions were at their worst (or best).

### Heart-rate variability indicators

We found strong associations between HR and HRV indicators, demonstrating the need to correct for HR when studying HRV, especially when HR differed between treatments as is the case in the present study. We found that HR increased when cows moved from the reference to the negative conditions, across all personality types. The increase in HR may reflect higher arousal and possibly higher levels of stress in these cows (Kovács *et al.* 2014). Furthermore, increased HR in the negative conditions may reflect a disturbance in sleeping patterns (and behaviour generally) as a result of limited access to cubicles. In line with this idea, a study in humans demonstrated that deviations from sleeping habits were associated with increased resting HR (Faust *et al.* 2020). Finally, this explanation also highlights a potential limitation of our study: while we did control for the effect of posture on HR data by focusing on recordings taken when cows were lying down, we did not control for the possible effects of sleep on our measurements. This may have influenced our results as HR was found to decrease with deepening sleeping stages in cows (Hunter *et al.* 2021). Future studies should therefore account for sleeping stages in addition to body postures when investigating the effects of affective treatments on HR.

Whether cows experienced the housing events positively or negatively remains impossible to determine on the sole basis of HR analyses. Numerous studies have demonstrated that HR can increase in response to both positive and negative affective states (Reefmann *et al.* 2009a; Briefer *et al.* 2015), but also in response to non-affectively charged events like locomotion – as a result of increased arousal.

When controlling for arousal (by controlling for HR), we found personality-dependent effects of housing conditions on HRV indicators. Inactive cows transitioned to the positive conditions had lower relative power of LF. From a psychological perspective, this finding suggests that these inactive cows were in a more positive state when housed in the positive conditions (compared to their reference housing) since positive states have been associated with lower relative power of LF in humans (Bhattacharyya *et al.* 2008). Non-active, non-fearful and non-social cows had higher relative power of HF in the positive housing, a result indicative of increased parasympathetic control (Shaffer & Ginsberg 2017). In animals, higher parasympathetic activity has been repeatedly associated with positive states and improved welfare (Reefmann *et al.* 2009b; Kowalik *et al.* 2017), while decreased parasympathetic activity has been associated with negative states and poorer welfare (Kovacs *et al.* 2015; Kowalik *et al.* 2017). Physiologically speaking, specific changes in the positive housing may thus have had relaxing effects that were positively perceived by certain cows depending on their personality profile. In particular, increased pro-social behaviours – as a result of enhanced social familiarity (Rault 2019) – and more frequent brushing may have led to an elevated parasympathetic activity, though we wonder whether an increase in pro-social behaviours would also apply to the less social cows. In line with this idea, previous studies in humans and other animals have demonstrated that massage, grooming and positive social connection were associated with increased parasympathetic activity (Kok *et al.* 2013; Grandi & Ishida 2015; Kowalik *et al.* 2017; Field 2019). Additional analyses of cow physiology and behaviour would, nonetheless, be necessary to validate our assumptions, since we also saw increases in HF in active cows in the negative housing. Analyses of plasma oxytocin in dairy cows, for instance, could be used to validate our theory since positive contacts appear to result in long-lasting oxytocin release (Rault 2016; Faraji *et al.* 2018). Moreover, analyses of time-budget and activity patterns of our focal cows would help us determine the extent to which our housing changes influenced the daily routine of the cows. We could, for instance, assess whether how the allocation of extra-space in the positive conditions affected social behaviour, and determine whether all cows (e.g. fearful and non-fearful) made use of the new brush. Such analyses could help us pinpoint events or housing changes that effectively challenged cows in a personality-dependent manner, positively or negatively.

Finally, and unexpectedly, we found that active cows in the negative conditions had higher SD1.SD2^-1^ in the negative compared to the reference or positive conditions – an index of parasympathetic HR modulation (Shaffer *et al.* 2014). Hence, active cows in the positive conditions appeared to have an altered parasympathetic control compared with active cows in the negative conditions. This physiological contrast may result from welfare and affect differences between the two subpopulations, since low SD1 measures have typically been associated with a wide range of psychological conditions such as depression, social anxiety disorders and post-traumatic stress disorders in humans (Kemp *et al.* 2012; Alvares *et al.* 2013; Meyer *et al.* 2016).

### Milk-derived indicators

The present study also investigated the effects of housing and personality on milk-derived indicators in dairy cows. We found MY to be reduced in the negative conditions, irrespective of personality. While a progressive decrease in MY is expected over the lactation period (Wilmink 1987), such a drop may also be indicative of stress (Rushen *et al.* 2001). This is in line with the higher HR also observed in the negative conditions.

Interestingly, fearful cows showed differences in milk yield fluctuations, specifically higher day-to-day fluctuations in the negative housing. Previous studies have linked daily milk fluctuations to disease and resilience and even suggest using this variable to breed more resilient and healthy cows (Elgersma *et al.* 2018; Wang *et al.* 2024). Milk yield fluctuations are easy to collect and monitor on farms that have individual, daily MY records per cow, and involve no additional handling. This indicator hence deserves to be investigated further in terms of its link to dairy cow welfare beyond simply disease, but in terms also of mental states.

The milk composition of fearful and social cows also differed according to the housing conditions. The milk of fearful and social cows moved to the negative conditions increased in relative fat content. Conversely, the fat content of the milk of non-fearful and non-social cows increased instead in the positive conditions. This result is not entirely aligned with previous studies, which revealed a negative relationship between cow resilience and relative fat content in milk (Poppe *et al.* 2021) and a drop in absolute fat content following acute stress (Hong *et al.* 2018). The discrepancy between our results and other findings may arise from underlying differences in terms of total MY and absolute milk composition. Based on measures of relative fat content solely, we cannot determine whether our relative fat increase originates from greater milk fat production or simply reflects a more concentrated milk (i.e. with less water). The fact that we observed increases in pFat in both the positive and negative housing compared to the reference could also point to a change linked to time in pregnancy and lactation. In the future, we recommend that researchers focus on absolute rather than relative measures of milk composition in order to identify whether housing conditions, and potential welfare differences, influence specific milk constituents. We found no effect of housing or personality on lactose. To our knowledge, only one study has investigated the effect of psychological stressors on lactose content in milk, in this case in humans, without finding any significant association between these two parameters (Ziomkiewicz *et al.* 2021). Research into the association between psychological factors and milk composition is still in its infancy and warrants further work.

### Study limitations

When it comes to identifying indicators of welfare (or affect) in non-verbal beings, we must consider the ‘sensitivity’ and ‘specificity’ of the indicator. Sensitivity is defined as how well and rapidly the indicator reflects changes in welfare (Walker *et al.* 2012). Low sensitivity occurs when minor changes in welfare do not translate to changes in the indicator, possibly because changes are delayed, or because changes require accumulations of positive or negative events over long periods of time (Walker *et al.* 2012). In the present study, with the experimental housing conditions being applied for only a period of six weeks, it is possible that stable changes in welfare were either minor (for example, short-term following each event), delayed, or not manifested in the physiology of the focal cows yet. Specificity is defined as how well an indicator distinguishes between positive and negative welfare states (Mason & Mendl 1993). Low specificity is observed, for example, in serum cortisol, which shows increases with both high arousal positive and negative states (Ede *et al.* 2019). If our physiological measures reflected primarily arousal, rather than valence, then these would be expected to show similar patterns in the positive and negative conditions, unless these cannot be considered to be inducing equal levels of arousal. Note that the present study did not investigate sensitivity or specificity as defined above, but we did attempt to assess and to some extent control for arousal by investigating HR.

### Animal welfare implications

The present work requires further research and does not lead to the direct creation of a tool that could be immediately used on-farm. Therefore, the direct implications of this work for dairy cattle welfare remain in our view somewhat limited. In addition, two of our three categories of indicators: hair cortisol and heart-rate variability, currently require a degree of extra handling of cows, which may be considered intrusive. If these indicators are to be used to monitor dairy cow welfare on-farm in an animal-friendly manner, further technology may first need to be developed. This is why we are especially enthusiastic about the milk-derived indicators, which can be routinely recorded on many dairy farms without further handling and may hence show potential in monitoring, and hopefully consequently improving, dairy cow welfare after further validation. Finally, we hope that these indicators will lead to further research in cattle and other species.

## Conclusion

In conclusion, we found that our negative housing involving a gradual worsening of the environment led to an increase in heart rate and a decrease in milk yield in primiparous dairy cows. Further research should investigate how milk varies with poor welfare across time. Furthermore, various indicators of heart-rate variability increased in the positive housing and decreased in the negative housing in many of the personality profiles. However, certain personality types showed opposing trends, with increases in heart-rate variability indicators in the negative housing conditions, specifically active personality types. This illustrates nicely how housing conditions can affect different cows differently. Further research in this area should investigate the impact of time in mediating the impact of housing changes on these physiological parameters, namely whether and how cows habituate to various changes and how long gradual changes need to be applied to lead to physiological changes.

## Supporting information

10.1017/awf.2026.10063.sm001 Kremer et al. supplementary material Kremer et al. supplementary material

## Supplementary material

The supplementary material for this article can be found at http://doi.org/10.1017/awf.2026.10063.
