# Supplementary material for: Non-invasive physiological indicators of welfare in dairy cows
Source: Anim Welf. 2026 Feb 5;35:e10. doi: 10.1017/awf.2026.10063 (PMC12895201; doi:10.1017/awf.2026.10063)
Supplement: Kremer et al. supplementary material [file S0962728626100633sup001.pdf]

Non-invasive physiological indicators of welfare in dairy cows: Supplementary material

Louise Kremer[1,2], Kees van Reenen[2], Akke Kok[3], Eddie AM Bokkers[1], Gerrit Gort[4], Jasper Engel[4], Joop TN van der Werfhttps://orcid.org/0000-0003-2341-7338[2] and Laura E Webb https://orcid.org/0000-0002-4943-4294[1]

[1] Animal Production Systems group, Wageningen University & Research, PO Box 338, 6700 AH Wageningen, The Netherlands

[2] Livestock Research, Wageningen University & Research, PO Box 338, 6700 AH Wageningen, The Netherlands

[3] Adaptation Physiology Group, Wageningen University & Research, PO Box 338, 6700 AH Wageningen, The Netherlands

[4] Biometris, Wageningen University & Research, PO Box 16, 6700 AA Wageningen, The Netherlands

Author for correspondence: Laura E Webb, email: laura.webb@wur.nl

Table S1. Number of dairy cows in each housing condition per personality trait. Cows were divided in two classes per personality trait based on their behavioural scores on the related personality trait in comparison to the median score. Superior (Sup.), Inferior (Inf.), Rotated component (RC), n number of cows per personality class in the reference conditions, n_1 number of cows per personality class in the positive conditions, n_2 number of cows per personality class in the negative conditions (from Kremer et al. 2021).

**Personality**			**Housing conditions (number)**		
**Trait (median score)**	**Class**	**Definition**	**Reference ($n = n_1+n_2$)**	**Positive (n_1)**	**Negative (n_2)**
RC1: Activity	Active	> -0.08	23	11	12
(-0.08)	Inactive	≤ -0.08	24	13	11
RC2: Fearfulness (0.23)	Fearful	≤ 0.23	24	13	11
	Non-fearful	> 0.23	23	11	12
RC3: Sociability (0.26)	Social	> 0.26	24	10	14
	Non-social	≤ 0.26	23	14	9
